# Integrated Diagnostics of Thyroid Nodules

**DOI:** 10.3390/cancers16020311

**Published:** 2024-01-11

**Authors:** Luca Giovanella, Alfredo Campennì, Murat Tuncel, Petra Petranović Ovčariček

**Affiliations:** 1 Department of Nuclear Medicine, Gruppo Ospedaliero Moncucco SA, Clinica Moncucco, 6900 Lugano, Switzerland; 2 Clinic for Nuclear Medicine, University Hospital Zürich, 8004 Zürich, Switzerland; 3 Nuclear Medicine Unit, Department of Biomedical and Dental Sciences and Morpho-Functional Imaging, University of Messina, 98100 Messina, Italy; alfredo.campenni@unime.it; 4 Department of Nuclear Medicine, Hacettepe University, 06230 Ankara, Turkey; murat.tuncel@hacettepe.edu.tr; 5 Department of Oncology and Nuclear Medicine, University Hospital Center Sestre Milosrdnice, 10 000 Zagreb, Croatia; p.petranovic@gmail.com; 6 School of Medicine, University of Zagreb, 10 000 Zagreb, Croatia

**Keywords:** thyroid, ultrasonography, thyroid stimulating hormone, nuclear medicine, cytopathology

## Abstract

**Simple Summary:**

Thyroid nodules are commonly detected in daily clinical practice, and their diagnosis and therapy usually involve different specialists and various diagnostic and therapeutic methods. Thyroid nodule management requires the integration of laboratory, imaging, and pathology examinations to achieve a proper diagnosis. It enables the elimination of unnecessary therapeutic procedures in many individuals and the timely identification of patients who require specific therapies. Furthermore, bioinformatics may change the current management of clinical data, enabling more personalized diagnostic approaches for patients with thyroid nodules. The clinical impact of artificial intelligence needs to be determined in further large-sample studies, especially in indeterminate cytology findings, that require “diagnostic surgery” to provide a definitive diagnosis.

**Abstract:**

Thyroid nodules are common findings, particularly in iodine-deficient regions. Our paper aims to revise different diagnostic tools available in clinical thyroidology and propose their rational integration. We will elaborate on the pros and cons of thyroid ultrasound (US) and its scoring systems, thyroid scintigraphy, fine-needle aspiration cytology (FNAC), molecular imaging, and artificial intelligence (AI). Ultrasonographic scoring systems can help differentiate between benign and malignant nodules. Depending on the constellation or number of suspicious ultrasound features, a FNAC is recommended. However, hyperfunctioning thyroid nodules are presumed to exclude malignancy with a very high negative predictive value (NPV). Particularly in regions where iodine supply is low, most hyperfunctioning thyroid nodules are seen in patients with normal thyroid-stimulating hormone (TSH) levels. Thyroid scintigraphy is essential for the detection of these nodules. Among non-toxic thyroid nodules, a careful application of US risk stratification systems is pivotal to exclude inappropriate FNAC and guide the procedure on suspicious ones. However, almost one-third of cytology examinations are rendered as indeterminate, requiring “diagnostic surgery” to provide a definitive diagnosis. ^99m^Tc-methoxy-isobutyl-isonitrile ([^99m^Tc]Tc-MIBI) and [^18^F]fluoro-deoxy-glucose ([^18^F]FDG) molecular imaging can spare those patients from unnecessary surgeries. The clinical value of AI in the evaluation of thyroid nodules needs to be determined.

## 1. Introduction

Thyroid nodules are more common in countries with iodine-deficient populations, and in women compared to men (ratio 4:1), and their prevalence increases with age and body mass index [1,2,3,4,5].

Luckily, most thyroid nodules (90% to 95%) are benign [6]. Risk factors for thyroid cancer include ionizing radiation (e.g., from cancer treatments, occupational exposure, or nuclear fallout, especially when the exposure occurs at a young age), rapid growth, hoarseness, and a family history of thyroid cancer or cancer syndromes (e.g., multiple endocrine neoplasia type 2, familial adenomatous polyposis) [7]. Notably, while thyroid nodules can be detected in up to 10% of healthy subjects by palpation, neck ultrasonography (US) may detect nodules in up to 68% of them, respectively [8,9,10]. Additionally, most thyroid nodules are currently detected incidentally (i.e., thyroid incidentalomas) when imaging procedures (i.e., computed tomography (CT), magnetic resonance imaging (MRI), and vascular Doppler) are performed for different indications [11]. Considering the high prevalence of thyroid nodules compared to the very low prevalence of thyroid malignancies, screening of thyroid cancer with neck US is discouraged as it results in overdiagnosis and overtreatment without improving patient outcomes [12]. Consequently, attending physicians are required to decide which nodules carry a significant risk of malignancy and require further diagnostic workup. Thyroid US scoring systems need to be integrated into daily clinical practice, complemented with the use of thyroid scintigraphy when indicated to avoid FNAC of low-risk and autonomously functioning nodules [13]. Furthermore, molecular imaging with [^99m^Tc]Tc-MIBI and [^18^F]FDG is not widely used nowadays, although its usefulness is clearly demonstrated in many studies [14,15,16,17]. It is highly recommended in indeterminate cytology findings to spare patients from “diagnostic” surgeries, improve their quality of life, and reduce total hospital costs caused by unnecessary procedures and their potential complications [14,15,16].

Our present narrative review aims to analyze the available literature concerning the diagnostic approach to thyroid nodules and provide an updated synopsis on the role of different procedures, including advanced molecular imaging. For this document, the authors volunteered to prepare the text for each section. A review of the literature was performed in PubMed, Web of Science, and Scopus without time or language restrictions through the use of one or more fitting search criteria and terms as well as through screening of references in relevant selected papers. The body of literature up to and including November 2023 was considered. Screening of titles/abstracts and removal of duplicates were performed and the full texts of the remaining potentially relevant articles that met the inclusion and exclusion criteria were retrieved and reviewed. Any disagreement was discussed until a consensus decision was reached.

Key learning points:

- Initial assessment of patients with thyroid nodules should be based on clinical history, clinical examination, and measurement of TSH level.
- Thyroid US and its scoring systems are important for the risk stratification of thyroid nodules.
- Thyroid scintigraphy is generally performed in patients with low to low-normal TSH value and nodules >1 cm in size.
- FNAC is performed for non-autonomous thyroid nodules, according to US scoring systems.
- [^99m^Tc]Tc-MIBI and [^18^F]FDG PET/CT are recommended in cytologically indeterminate thyroid nodules to reduce “diagnostic” surgeries.
- The clinical value of AI in the evaluation of thyroid nodules needs to be determined.

## 2. Clinical History and Clinical Examination

The initial assessment of individuals with thyroid nodules detected by palpation or during radiologic procedures includes clinical history and examination, measurement of serum TSH level, US of thyroid nodules and neck lymph nodes, thyroid scintigraphy (Na[^99m^Tc]TcO_4_ or Na[^123^I]I) in cases of suppressed or low-normal TSH values, and FNAC if indicated according to US findings and thyroid scintigraphy.

### 2.1. Clinical History

Clinical history suggestive of increased risk of malignancy includes rapid nodule enlargement, head and neck external beam radiation therapy (particularly during childhood), whole-body irradiation (e.g., before bone marrow transplantation), and family history of thyroid cancer or thyroid cancer syndromes [18]. Familial occurrence of DTC is demonstrated in 5–10% of cases [19,20,21]. Some studies revealed that DTC occurs earlier [19,22], in a more advanced stage and more aggressive [23] with a worse outcome in the next generation [19]. Park et al. demonstrated a higher DTC recurrence rate in familial cases compared with sporadic ones and the second generation had more aggressive clinical features compared with the first one [24]. Therefore, patients with familial DTC need a careful clinical history evaluation. Thyroid cancer syndromes should be considered as they demand screening of different components of the syndrome in first-degree relatives. They include multiple endocrine neoplasia type 2 (MEN 2), Cowden syndrome, familial adenomatous polyposis, Werner syndrome, and Carney complex. Approximately 0.1% of all DTC cases are associated with these syndromes, except MEN2A and MEN2B, where the rates are 0.3% and 0.2%, respectively [25].

### 2.2. Clinical Examination

Thyroid gland palpation is usually the first clinical examination; however, it has low sensitivity (2–6%) for detecting thyroid nodules [26] and, in some cases, the physical examination may be limited by body habitus [27]. Most malignant thyroid nodules are asymptomatic, and a great majority of patients are euthyroid. However, there are several clinical examination findings harboring a higher risk of cancer, i.e., fixed and firm nodules, nodules larger than 4 cm in size (19% incidence of malignancy) [28], cervical lymphadenopathy, symptoms of obstruction, dysphonia, and vocal cord paralysis [6,27]. Furthermore, a combination of a solitary nodule, cervical lymphadenopathy (>1 cm), and vocal cord paralysis has a positive predictive value (PPV) of almost 100% for malignancy [29].

## 3. Thyroid Laboratory, Imaging, and Cytopathology

### 3.1. Laboratory Medicine

Thyroid function can be accurately assessed by measuring TSH and free thyroid hormones (i.e., free thyroxine, FT4; free tri-iodo-thyronine, fT3). TSH and FT4 have a complex, non-linear, inverse relationship resulting in relatively large changes in TSH compared to small changes in FT4 concentrations, respectively [30,31,32]. Accordingly, except in some rare conditions (i.e., central hypothyroidism, resistance to thyroid hormones, TSH-secreting pituitary adenoma, hyperthyroidism under treatment, and euthyroid sick syndrome), TSH measurement is a sensitive and the most accurate test for thyroid dysfunction [33,34]. As a consequence, different guidelines endorse the measurement of TSH alone at the front line while restricting FT4 (and rarely FT3) measurement in cases with abnormal TSH results (i.e., TSH reflex strategy) [35,36,37,38]. The same strategy is recommended in patients with thyroid nodules where TSH measurement is unanimously recommended as the first-line functional test by available clinical guidelines.

In patients with thyroid nodules, low TSH levels may be related to autonomously functioning thyroid nodule(s) and thyroid scintigraphy is indicated. A normal TSH excludes a clinically significant autonomy but, especially in countries with low iodine intake, cannot exclude compensated autonomy: in those regions, thyroid scintigraphy may properly exclude such nodules (frequently suspicious at neck US) from inappropriate FNAC [13,39]. Routine measurement of serum anti-thyroid peroxidase (TPO) antibodies is not necessary for thyroid nodule evaluation [10,40] and routine measurement of serum thyroglobulin (Tg) is strongly discouraged as it may be elevated in different thyroid diseases, including benign ones, and is aspecific and relatively insensitive for thyroid cancer [41]. Calcitonin is the standard biochemical tumor marker for medullary thyroid carcinoma (MTC) diagnosis and follow-up [42]. However, the value of routine testing in patients with thyroid nodules remains questionable due to the low prevalence, which results in a low PPV of basal calcitonin testing. Indeed, whether routine calcitonin testing improves prognosis in MTC patients remains unclear [43].

### 3.2. Thyroid Ultrasound

Since the 1970s, thyroid US has progressively gained a central role in assessing thyroid diseases. High-resolution US examinations are widely used worldwide, being radiation-free, relatively cheap, easy to learn, and versatile compared to other imaging modalities. Ultrasound devices are equipped with transducer probes with variable frequency (i.e., 2–20 Mega Hertz (MHz)). High-resolution linear transducers with a 7–15 MHz frequency are currently employed for thyroid examination. Since the thyroid gland is superficially located with its posterior border generally situated less than 4 cm from the skin, high-resolution (≥12 MHz) probes provide excellent image quality. High-resolution conventional B-mode (i.e., gray-scale ultrasound) evaluation is now integrated with multiparametric ultrasound (MPUS), including vascularization assessment (spectral Doppler, SD; color Doppler, CD; power Doppler ultrasound, PD; superb microvascular imaging, SMI; contrast-enhanced ultrasound, CEUS) and tissue stiffness assessment (sonoelastography), respectively [13].

In clinical practice, US is the first-line imaging method for the examination of thyroid morphology and structure. The main indications of thyroid US are summarized in Table 1.

Although US is critical for the evaluation of diffuse thyroid disease, differentiating between benign and malignant nodules is the main application area. Despite several ancillary techniques like elastography and Doppler that were proposed to differentiate malignant nodules from benign ones, the most commonly used parameters are the high-resolution B-mode ultrasound characteristics of thyroid nodules. Sonographic findings, including assessment of the nodule echogenicity, internal composition, calcification, and border regularity, are commonly used for differential diagnosis. Briefly, solid, hypoechoic nodules, taller-than-wide shape, and irregular borders with microcalcifications have the highest chance of being malignant [44].

Today, thyroid US allows an accurate evaluation of morphologic features, which have been used to propose a standardized risk stratification for thyroid nodules (i.e., Thyroid Imaging And Data Reporting Systems (TI-RADS)) attempting to reduce the admittedly high inter-operator variability [13]. Among these, the American College of Radiology (ACR)-TI-RADS, European (EU)-TI-RADS, Korean (K)-TI-RADS, British Thyroid Association (BTA), American Thyroid Association (ATA) classification and American Association of Clinical Endocrinologists (AACE), American College of Endocrinology (ACE), and Associazione Medici Endocrinologi (AME) classification systems are commonly used [18,45,46,47,48,49]. The rationale of these classification systems is that the risk of malignancy rises in parallel with the increase in the number of suspicious US features and the lack of benign findings. Risk classification aims to identify the most clinically significant malignancies and decrease the number of unnecessary FNACs on benign nodules. Reviewing all the classification systems is beyond the article’s scope, but we will briefly mention the most commonly used ACR-TIRADS, EU-TIRADS, and ATA thyroid ultrasound risk stratification systems (RSS).

American College of Radiology (ACR)-TI-RADS [45] is based on the assessment of different US features of thyroid nodules: composition (spongiform, mixed cystic, and solid), echogenicity (anechoic, hyperechoic/isoechoic hypoechoic and very hypoechoic), shape (wider than tall or taller than wide), margin (smooth, ill-defined, lobulated or irregular, extra-thyroidal), and echogenic foci (none or large comet-tail artifacts, macrocalcifications, peripheral (rim) calcification, and punctate echogenic foci). It associates each of these features with a score ranging from 0 to 3 points. The sum of the assigned points defines the risk of malignancies according to five grades, with each grade corresponding to benign (TR1: 0 points), not suspicious (TR2: 2 points), mildly suspicious (TR3: 3 points), moderately suspicious (TR4: 4–6 points), or highly suspicious for malignancy (TR5: ≥7 points). This system does not include subcategories or a TR0 group to indicate a normal thyroid. EU-TIRADS is one of the most commonly used TI-RADS systems across Europe. EU-TIRADS 1 defines a normal thyroid gland without nodules. EU-TIRADS 2 is defined as a benign category, whereas EU-TIRADS 3 defines a nodule with a low risk of malignancy. Nodules with EU-TIRADS 4 have an intermediate risk, and with EU-TIRADS 5 have a high risk of malignancy. Detailed EU-TIRADS categories, risks of malignancy, and recommendations are explained in Table 2.

The American Thyroid Association (ATA) guidelines for assessing thyroid nodules are meant to improve inter- and intra-reader consistency when reporting thyroid nodules on ultrasound and facilitate communication with referring physicians. The 2015 guideline emphasizes the importance of the sonographic pattern of the nodule for risk stratification. This system does not include scoring but categorizes the risk of malignancy from very low risk to high. The malignancy risk as well as the size of the nodule are the two main criteria for FNA (Table 3).

**Figure 1:**
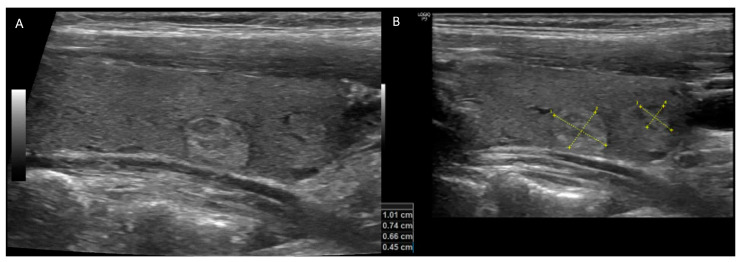
A 56-year-old woman with small hyperechoic and isoechoic nodules with regular borders ((**A**) longitudinal-plane US image) and size equal to and less than 1 cm ((**B**) labeled longitudinal-plane image). Thyroid US risk evaluations were reported using ACR TIRADS 3, EU-TIRADS 3, ATA classifications: low suspicion. Considering the size and risk of the nodules, no biopsy was recommended.

Comparison of thyroid ultrasound RSS is an ongoing debate in the literature. This is understandable; each society deems its RSS to be the preferred system. Endocrinologists from Europe prefer EU-RADS, endocrinologists from the USA uses ATA guidelines, and radiologists and nuclear medicine physicians prefer the ACR-TIRADS system [50,51] (Figure 1).

**Figure 2:**
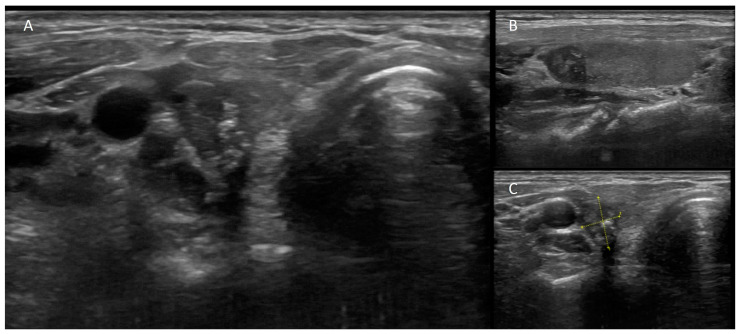
A 45-year-old woman with a family history of thyroid cancer. US showed ((**A**) transverse-plane image, (**B**) longitudinal-plane image, (**C**) labeled longitudinal-plane image) hypoechoic nodule with microcalcifications, taller-than-wide shape, irregular borders, and size > 1 cm. Thyroid US risk evaluations were reported using ACR TIRADS 5, EU-TIRADS 5, and ATA classifications: high suspicion. Considering the size and risk of the nodules, FNAC was performed; they were reported to be malignant, and total thyroidectomy revealed papillary thyroid carcinoma.

In a multicentric German trail, EU-TIRADS was proved to be inferior when compared to other RSSs with diagnostic accuracies of 0.70 vs. 0.79, 0.78, 0.82, and 0.79 for Kwak-TIRADS, ACR-TI-RADS, Korean-TIRADS, and American Thyroid Association (ATA) Guidelines, respectively [52]. In another study, authors found that ACR TI-RADS, American Association of Clinical Endocrinologists/American College of Endocrinology/Associazione Medici Endocrinologi guidelines, European TI-RADS, ATA guidelines, and Korean TI-RADS would have avoided FNA for 34.7%, 31%, 25.7%, 20%, and 6% of nodules with false-negative rates (FNRs) of 24%, 28.5%, 22%, 7.2%, and 1.9%, respectively. In this study, ATA guidelines had the highest area under the curve and a low FNR, whereas ACR TI-RADS would have spared more patients from FNA with a high FNR [53]. So far, no uniform, worldwide accepted RSS has been established because of the controversy in the literature, and the differences in the expertise and preferences of ultrasonographers (Figure 2). Also, there are limitations of RSS like insufficient sensitivity for the diagnosis of follicular thyroid carcinoma and follicular subtypes of PTC and insufficient specificity to rule out autonomously functioning thyroid nodules from FNAC. However, work has recently begun on a new international US-based RSS for thyroid nodules. With the participation of several scientific societies, an International TIRADS (I-TIRADS) will be proposed and established internationally as a uniform evidence-based system. Currently, several working groups are investigating ultrasound criteria. In addition, promising data already exist regarding the use of AI to identify US patterns. This technique could significantly reduce interobserver variability and may be associated with improvements in specificity and accuracy, without significantly sacrificing sensitivity for malignancy detection [54].

### 3.3. Nuclear Medicine

**Figure 3:**
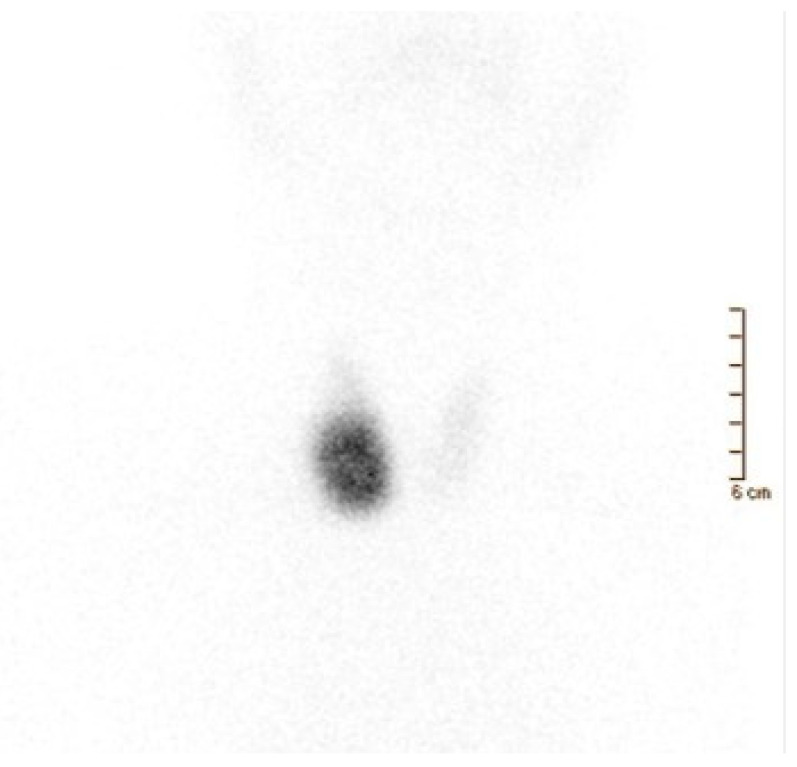
A 65-year-old man affected by overt hyperthyroidism (TSH 0.00 μIU/mL (0.35–4.94), FT3 9.61 pg/mL (1.58–3.91), FT4 2.25 ng/dL (0.70–1.48)) associated with tachycardia, insomnia, and irritability. Thyroid ultrasonography showed a large-sized nodule (38 × 34 × 20 mm) in the right thyroid lobe, isoechoic with a hypoechoic ring. Moderately increased intra-nodular blood flow was also noted. Thyroid scintigraphy was performed 20 min after Na[^99m^Tc]TcO_4_ administration (111 MBq). Image (anterior view; magnification: 1.4; matrix: 256 × 256; time frame: 100 Kcs) demonstrated a well-defined hyperfunctioning area located in the middle–lower part of the right thyroid lobe. In this latter, tracer uptake/distribution was quite intense and homogeneous, respectively. On the contrary, mild tracer uptake was noted in the remaining right lobe (upper part), isthmus, and left lobe. Thus, a toxic nodular goiter with severe functional inhibition of the remaining thyroid parenchyma was diagnosed.

Thyroid scintigraphy performed with functional tracers is used to map the global and regional activity of sodium iodide transporter (NIS) within the thyroid gland. Nowadays, it is commonly performed by using Na[^99m^Tc]TcO_4_, preferred over iodine tracers (Na[^131^I]I or Na[^123^I]I) due to its shorter physical half-life (6 h), pure gamma emission (140 keV), low radiation burden, wide availability, and significantly lower costs [55]. Na[^99m^Tc]TcO_4_ is caught within thyrocytes through NIS located on the basolateral membrane, like radioiodine tracers. On the contrary, as the main difference, it is not organified and leaves thyrocytes with an effective half-life (6 h) shorter than that of Na[^123^I]I (about 13 h) and Na[^131^I]I (about 8 days). However, Na[^99m^Tc]TcO_4_ uptake is representative of thyroid hormone biosynthesis and provides relevant clinical information on the global and regional function of thyroid cells (Figure 3).

In physiological conditions, NIS activity is positively regulated by TSH and, as a consequence, Na[^99m^Tc]TcO_4_ uptake in the gland can be properly interpreted in light of TSH values (ideally performed before thyroid scintigraphy). The activity of NIS is inversely regulated by the intrathyroidal iodine pool that reflects the iodine intake.

Although almost all thyroid cancers are non-functioning, most of these nodules are benign (i.e., 90–95%), which greatly reduces the specificity of a thyroid scan. Accordingly, a thyroid scan is generally performed when nodules occur in people with low or low-to-normal TSH levels. The relationship between thyroid autonomy and TSH levels, however, is affected by the degree of iodine sufficiency and varies widely regionally [31,32,34]. Therefore, although autonomous nodules are almost invariably accompanied by decreased TSH levels (i.e., <0.1–0.4 mUI/L) when the iodine supply is adequate, the bulk of autonomous tissue may be insufficient to suppress the TSH level in iodine-depleted thyroids, especially in the early phases of autonomy. As a consequence, different indications are given in current clinical guidelines with a thyroid scan recommended when the TSH level is low or low to normal in the USA (iodine-repleted country), whereas a Na[^99m^Tc]TcO_4_ scan is recommended in all people with a nodule greater than 10 mm, independently of the TSH level in Germany (iodine-deficient country) [5,39,55,56]

**Figure 4:**
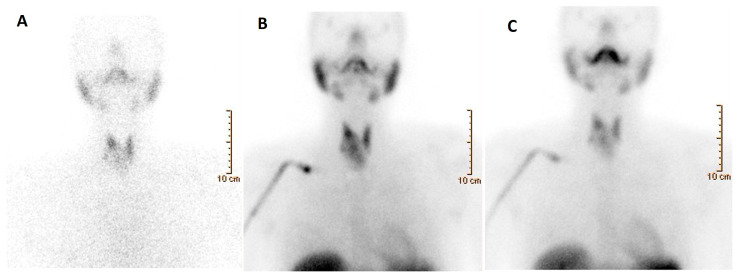
A 52-year-old woman affected by nodular goiter in euthyroid status (TSH 0.60 μIU/mL (0.35–5.50), FT3 3.18 pg/mL (2.30–4.20), FT4 1.17 ng/dL (0.89–1.76)). Thyroid ultrasonography showed a large-sized nodule (39 × 36 × 33 mm) in the right thyroid lobe, irregularly hypoechoic with the presence of isoechoic areas. A moderately increased intra-nodular blood flow was also noted. (**A**) Thyroid scintigraphy, performed 20 min after Na[^99m^Tc]TcO_4_ administration (111 MBq; anterior view; magnification: 1.4; matrix: 256 × 256; time frame: 100 Kcs), showed a well-defined hypofunctioning area located in the middle–lower part of the right thyroid lobe. Thus, a nodular goiter was diagnosed while FNAC was conclusive for a benign lesion (Tir2) according to the Italian scoring system. [^99m^Tc]Tc-MIBI scintigraphy was obtained 10 and 120 min (**B**,**C**, respectively) after tracer administration (370 MBq). Images were acquired in anterior view, magnification 256 × 256, matrix 1.4, time frame 10 min. Increased [^99m^Tc]Tc-MIBI uptake was noted in the hypofunctioning nodule.

**Figure 5:**
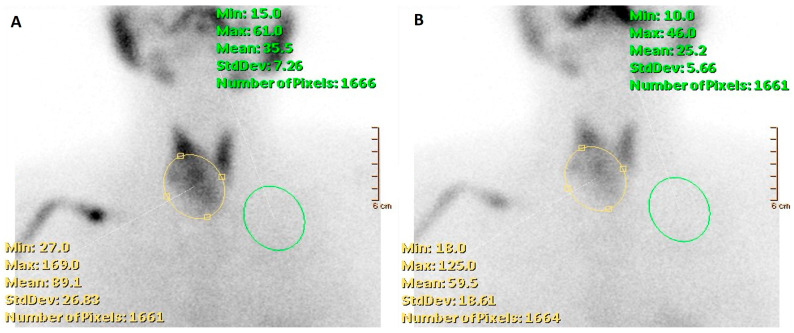
A 52-year-old woman affected by nodular goiter, cytologically benign (TIR2 according to the Italian scoring system). [^99m^Tc]Tc-MIBI scintigraphy was obtained 10 and 120 min after tracer administration (**A**,**B**, respectively) using a semiquantitative approach by calculating the wash-out index (WOind). WOind value was <−19% (exactly −37%), thus consistent with a no-malignant lesion. Final histology diagnosis: follicular adenoma.

Consequently, high iodine concentrations (e.g., regular use of products rich in iodine) may reduce the quality of a functional image. For this reason, any “excess” iodine intake should be avoided before scintigraphy or it should be postponed for several (i.e., 1–3) to many (i.e., 6 or more) months in patients with severe iodine contamination due to radiological contrast media administration or amiodarone therapy, respectively [55]. [^99m^Tc]Tc-MIBI is a lipophilic cation able to cross the cell membrane, reversibly penetrating the cytoplasm and then irreversibly moving through the membrane of the mitochondria using a different electrical gradient caused by a high negative electric potential of the inner membrane. It is regularly used as a marker for myocardial perfusion and hyperfunctioning parathyroid tissue. All in all, studies reported its abnormal uptake in different tumors such as in the lungs, brain, breast, bone, and thyroid [57,58,59,60]. The role of [^99m^Tc]Tc-MIBI thyroid scintigraphy (i.e., molecular imaging) to better assess the risk of malignancy in hypofunctioning and cytologically indeterminate thyroid nodules has been investigated for more than three decades [56]. In 2004, Hurtado-Lopez first compared intranodular [^99m^Tc]Tc-MIBI and Na[^99m^Tc]TcO_4_ uptake and found a quite absolute NPV for nodules with a matching hypoactive pattern with both tracers [61]. More recently, the [^99m^Tc]Tc-MIBI uptake in the thyroid nodule has been qualitatively (i.e., visual analysis) compared to the uptake in the extranodular (i.e., normal) thyroid parenchyma and classified as hyper-, iso-, and hypointense, with the latter ruling out malignancy with up to 99% NPV [62,63]. Vice versa, an increased [^99m^Tc]Tc-MIBI uptake with respect to surrounding parenchyma or compared to a Na[^99m^Tc]TcO_4_ image conferred a higher risk of cancer [61,62,64,65,66]. However, a positive pattern (i.e., abnormal [^99m^Tc]Tc-MIBI uptake in the nodule) was found in a significant proportion of histologically benign nodules (especially follicular and oxyphilic adenomas), and an unsatisfactory PPV (i.e., 27%) was reported [67]. To improve the diagnostic performance of molecular imaging in such patients, Saggiorato and colleagues proposed a semiquantitative approach using the so-called Retention Index (RI) method [68]. They concluded that [^99m^Tc]Tc-MIBI-positive nodules with an RI value ≥ −11.94 were suspicious for malignancy, thus suggesting more aggressive clinical management. In addition, they proved a reduced accuracy of [^99m^Tc]Tc-MIBI scintigraphy in oxyphilic nodules and discouraged its use in such a setting. More recently, a new semiquantitative method (i.e., wash-out index—WO*ind*) was proposed, taking into account the [^99m^Tc]Tc-MIBI kinetics within the nodules, and was found able to improve the diagnostic accuracy compared to qualitative evaluation [63,64,66] (Figure 4 and Figure 5).

Finally, Schenke and colleagues, in a recent European multicenter study, confirmed that the semiquantitative approach using the WO*ind* method could significantly improve the overall diagnostic performance of [^99m^Tc]Tc-MIBI imaging [65]. In clinical routine, thyroid [^99m^Tc]Tc-MIBI imaging can also be used to differentiate between amiodarone-induced hyperthyroidism (AIT) type 1 (i.e., normal or high uptake) and type 2 (low uptake), respectively [69,70].

Currently, single-photon emission computed tomography (SPECT) associated with computed tomography (SPECT/CT, hybrid imaging) is able to provide a co-registration of anatomic and functional/molecular data results for a better localization and characterization of tracer uptake [65]. [^18^F]FDG positron emission tomography/computed tomography (PET/CT) is widely used for initial staging, restaging, recurrence detection, and assessment of treatment outcomes in a variety of malignant diseases [71]. [^18^F]FDG uptake is related to an overexpression of the transmembrane glucose transporter proteins (GLUTs), which move the tracer into the cell, and to the overactivation of hexokinases that phosphorylate [^18^F]FDG to [^18^F]FDG-6-phosphate and trap the tracer in the cell [72]. Interestingly, a visually [^18^F]FDG-negative thyroid nodule with indeterminate cytology carries a negligible risk of malignancy, making [^18^F]FDG an accurate ruling-out biomarker. Notably, a recent blinded, randomized, controlled multicenter trial in the Netherlands consistently proved that a [^18^F]FDG PET/CT-driven workup of cytologically indeterminate thyroid nodules may change the clinical management and safely reduce inappropriate surgical interventions by 40%. Notably, the authors warned against the use of [^18^F]FDG PET/CT in patients with Hurthle cell nodules where a high [^18^F]FDG is expected, even in benign ones [73].

**Figure 6:**
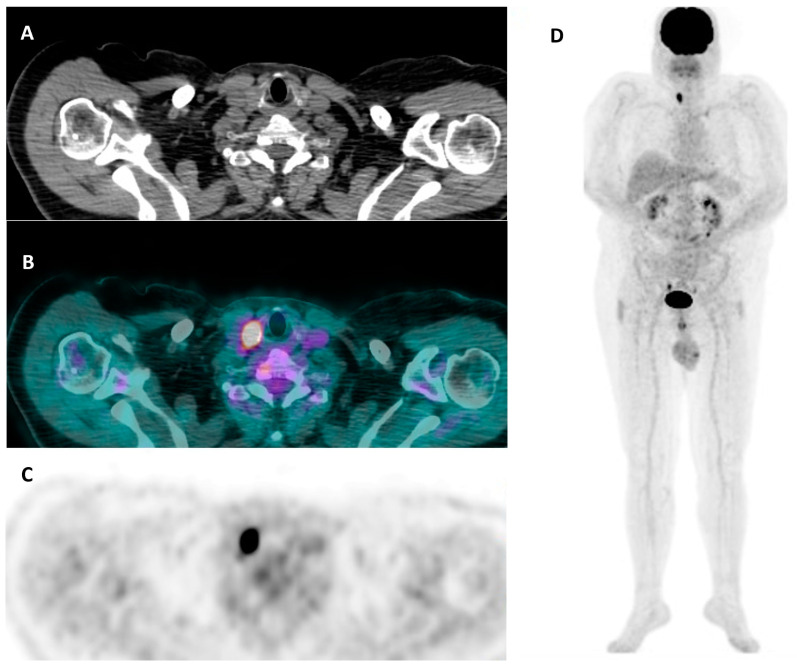
A 62-year-old man with a hypermetabolic thyroid nodule (SUV max = 18.3) unexpectedly discovered in the right lobe during a [^18^F]FDG PET/CT study performed for staging in suspected paraneoplastic syndrome. (**A**) CT – axial view, (**B**) a [^18^F]FDG PET/CT axial view, (**C**) [^18^F]FDG PET axial view, (**D**) whole-body [^18^F]FDG PET. A laboratory test was consistent with euthyroid status (i.e., TSH 2.1 µIU/mL (0.27–4.2), FT3 3.30 pg/mL (2.0–4.4), FT4 18.2 pmol/L (12–22)) with negative anti-thyroid antibodies (i.e., TPOAb, TgAb). Thyroid ultrasound demonstrated a multinodular goiter with a dominant hypoechoic nodule in the right lobe (maximum size: 22 mm), which was then classified as hypofunctioning at thyroid scintigraphy. Accordingly, FNAC was performed: an indeterminate lesion with a high risk of being malignant (i.e., TIR3B according to the Italian scoring system) was diagnosed. The patient underwent total thyroidectomy, and the final histological diagnosis was consistent with Hurtle cell follicular adenoma.

Conversely, a positive [^18^F]FDG PET/CT is not reliable as a rule-in test since approximately 50% of patients with positive nodules have a benign disease in the final histological report [74,75,76] (Figure 6).

In summary, either [^99m^Tc]Tc-MIBI or [^18^F]FDG PET/CT may safely rule out inappropriate diagnostic surgeries in about half of patients with cytologically indeterminate nodules (non-Hurthle types). When adopted as a rule-out test, both [^99m^Tc]Tc-MIBI scintigraphy and [^18^F]FDG PET/CT also proved to be cost-effective in comparison with the standard practice (i.e., diagnostic surgery) and the use of Gene Expression Classifiers [14,15,16,17]. Their use, however, should be optimized and restricted to patients with non-Hurtle cell cytological patterns and without additional indications to surgery as multinodular goiters or large-index lesions (i.e., >40–50 mm). However, more specific rule-in biomarkers are still warranted in patients with [^99m^Tc]Tc-MIBI- or [^18^F]FDG-positive cytologically indeterminate nodules in order to improve the PPV.

Moreover, radiomics analysis of [^18^F]FDG PET/CT data preliminarily proved to increase specificity and PPV in discriminating benign from malignant cytologically indeterminate nodules [77]. However, currently, available data are contrasting [78], likely due to differences in pretest probabilities and other variables. All in all, the application of radiomic analysis in this setting should be reserved for clinical studies and not performed to make clinical decisions.

### 3.4. Fine-Needle Aspiration Cytology and Cytopathology

Thyroid nodules are common in clinical practice, with a prevalence of up to 60% depending on age, sex, etc. [3,4,26]. The majority are benign [79], and the risk of malignancy is 7 to 15% [79], depending on the nodule size, findings of ultrasound and nuclear medicine techniques, and patient characteristics.

Several guidelines and RSSs (Table 4) recommend biopsy based on the size and imaging findings.

The shape, margin, echogenicity, and presence of calcification are useful criteria for the discrimination of malignant from benign nodules [80].

FNAC Technique:

- *Preprocedural*

The patient should be informed that limited intra- and peri-thyroidal bleeding and mild local pain radiating to the ear may occur. Informed consent should be obtained after a detailed procedure discussion with the patient. The most significant possible complication of the procedure is the development of a neck hematoma, especially in hypervascular nodules, but this complication is fortunately rare [81] and usually resolves without intervention. A preprocedural test for coagulation is not routinely needed, but the patient should be questioned about recent or current anticoagulant therapy. To avoid excessive bleeding, anticoagulation therapy should be discontinued 4–7 days before biopsy; however, the preprocedural discontinuation of aspirin therapy is controversial [82]. Most use fine or thin (22- to 27-gauge) needles for FNAC. Thinner needles should be preferred for hypervascular nodules. The procedure can be performed with local anesthesia (lidocaine hydrochloride 1–2%, 1–2 mL) or without. Oertel and colleagues advocated using ice as an alternative for local anesthesia because it numbs the area and causes vasoconstriction, decreasing the aspirate’s hemodilution [83]. Per nodule, three passes are typically obtained, particularly if rapid on-site evaluation by a cytopathologist is unavailable. Under US guidance, the needle may be introduced parallel or perpendicular to the transducer, and the needle tip should be carefully monitored during the procedure. The collected material is placed on glass slides, smeared, and fixed in 95% ethyl alcohol or left to air dry.

- *Specimen Staining*

When using the Papanicolaou staining method, the smears should be quickly placed in 95% ethyl alcohol. When Diff-Quik or Giemsa stain is used, the smear should be allowed to air dry. Papanicolaou staining is most commonly used for cytologic analysis of thyroid specimens, and it provides the most precise depiction of nuclear chromatin, ground-glass nuclei, and nuclear groove characteristics in papillary carcinoma. Diff-Quik or Giemsa stain helps visualize the characteristics of the cytoplasm and colloid [83].

- *Material Adequacy and False-Negative Results*

Appropriate specimen preparation significantly increases the likelihood of material adequacy and decreases the frequency of false-negative findings. Both may be affected by the level of operator experience, lesion localization, method of guidance (palpation or US), number of aspirations, needle gauge, sampling technique, capability for immediate on-site cytologic analysis, and many other factors. The percentage of unsatisfactory specimens should remain less than 15%, or, ideally, 10%.

- *Comparison of Aspiration and Capillary Action*

Thyroid fine-needle aspiration is a safe, simple, relatively accurate, and first-line diagnostic tool in the evaluation of thyroid nodules. However, especially in hypervascular lesions, the aspiration technique frequently leads to microscopic hemorrhages, which obscure proper cytologic interpretation. Fine-needle capillary (FNC) sampling, a technique without aspiration, was developed in the 1980s [84]. It has been suggested that this non-aspiration sampling technique could reduce the amount of blood in samples and produce superior-quality specimens. Various comparative studies showed that aspiration FNAC and FNC sampling have no statistically significant difference in sample adequacy and diagnostic accuracy. The choice of technique should be based on the operator’s personal preferences and experience. However, Degirmenci and colleagues showed that selecting finer needles (24–25 G) for sonography-guided sampling of thyroid nodules and using the FNC technique increased the rate of adequate material in the cytological examination when compared to aspiration FNAC (76.9% vs. 49.4%, *p* < 0.001) [85]. Non-aspiration FNC sampling is less traumatic and causes less hemodilution of aspirate, which is critical for hypervascular lesions. Titton et al. and Oertel have suggested that the thyroid sampling should begin with non-aspiration FNC and, if the specimens collected with that technique are inadequate, should continue with aspiration FNAC [83,86].

- *Comparison with Core-Needle Biopsy*

Core-needle biopsy is performed with an 18–20-gauge needle and provides a large histologic tissue core, which may have a greater effect on surgical decision-making than cytologic diagnosis. In a separate prospective study in which FNAC was compared with core-needle biopsy performed with a spring-activated, short-throw, 18–20-gauge needle, the diagnostic yield with core-needle biopsy of thyroid nodules exceeded that with FNAC techniques by approximately 10% [87]. As can be predicted, there was also a higher complication rate with the use of the core needle and a higher need for anesthesia [88]. For these reasons, a core-needle biopsy is considered for patients in whom FNAC produces only specimens of inadequate cellularity after several passes or in patients who return for a repeat biopsy after a non-diagnostic initial FNAC.

Fine-needle aspiration cytology represents the diagnostic cornerstone because of its accuracy, reproducibility, and cost-effectiveness. However, FNAC is characterized by a grey diagnostic area in which the indeterminate cytology precludes a distinction between benign and malignant lesions. Physicians should manage the patients according to the different findings of cytology reports concerning the chance of malignancy. Several reporting systems and recommendations were proposed. Among these reporting systems, the Italian SIAPeC-AIT classification, in its latest version updated in 2014; The Bethesda System for Reporting Thyroid Cytopathology, proposed in 2007 and updated in 2017 and 2023; and the Guidance On The Reporting Of Thyroid Cytology Specimens from The UK Royal College Of Pathologists (RCPath) are among the most widely used in the world [89,90,91,92].

The Bethesda System for Reporting Thyroid Cytopathology is preferred by many centers, especially in the USA. The updated version (Table 5) also accounts for the possibility of a relatively new entity: non-invasive follicular neoplasm with papillary-like nuclear features (NIFTP).

The system includes six categories, with the most controversial being III–IV, where repeat FNAC, molecular testing, or lobectomy may be warranted. The cytological characteristics of the indeterminate categories (III–IV) can be divided into four different subgroups: qualitatively unsatisfactory specimen, cytologic atypia (mostly nuclear), architectural atypia (microfollicular), and the combination of the latter two [94]. The first case includes specimens with inadequate preparation and artifacts that may cause the appearance of nuclear enlargements suspicious for atypia. Specimens with atypical cells also belong to this category. Atypia can be epithelial, lymphoid, mesenchymal, or even non-specific and does not suggest any specific tumor by itself (Bethesda III). The second case includes cytological atypia in specimens, no matter the cell architecture. Usually, there are very few atypical cells, so the confirmation/exclusion of malignancy is not possible. The presence of oncocytes, without an adequate clinical picture, e.g., Hashimoto’s thyroiditis, is also considered atypical (Bethesda III). The third case involves specimens with some or abundant microfollicular structures but without atypia of the nucleus (Bethesda III and IV). Also, samples with many oncocytes, but without colloid or lymphocytes, are in this category (Bethesda IV). The fourth case includes scarce cellularity and the presence of microfollicular structures and nuclear atypia (Bethesda III). FNAC in these cases is considered as a screening tool because it cannot confirm or exclude malignancy [94].

Although the morphologic parameters used by these various systems (Table 6) are very similar, there are differences in the criteria for inclusion in cytologically indeterminate categories which may affect the recommendations for the clinical management of patients (Table 7).

Despite all these nuances in ultrasound RSS and cytopathology reporting systems, physicians should decide the optimal management with the help of the patient’s history and preferences, the data obtained from nuclear medicine imaging findings, and molecular testing if available.

## 4. Molecular Biomarkers

Molecular markers may have diagnostic or prognostic purposes. Approximately 15 to 30% of thyroid nodules are classified as indeterminate in the FNAC report [95]. They include Bethesda III (atypia of undetermined significance) and Bethesda IV (follicular neoplasm) lesions, with risks of malignancy (mean, range) of 28 (11–54)% and 50 (28–100)%, respectively [92]. If excluding nodules diagnosed by surgical pathologic examination as non-invasive follicular thyroid neoplasms with papillary-like nuclear features, the risks of malignancy are 6.4 (6–20) and 7.1 (0.2–30) [92]. The principal use of molecular markers in this FNAC categories is diagnostic, i.e., ruling out or ruling in thyroid cancer, with implications for further patient management.

The Cancer Genome Atlas Research Network 2014 investigated the molecular profile of 496 papillary thyroid cancers (PTCs), mainly classical and follicular subtypes, and demonstrated two distinct molecular profiles: BRAF^V600E^-like and RAS-like [96]. The first included the BRAF^V600E^ mutation as well as RET/PTC and BRAF fusions, while the other one included RAS family (HRAS, NRAS, KRAS) mutations, BRAF^K601E^ mutation, EIF1AX mutations, and THADA and PPARG fusions. These two molecular profiles seem to be associated with classical and follicular PTCs but are also noted in other DTC histotypes. Non-invasive follicular neoplasm with papillary-like nuclear features (NIFTP) and follicular thyroid carcinoma (FTC) are also considered RAS-like tumors [95]. Furthermore, benign lesions may also have RAS-like molecular profiles [97].

Molecular panels for indeterminate thyroid nodules range from the 7-gene panel, including BRAF, KRAS, NRAS, HRAS, RET/PTC1, RET/PTC3, and PPARG/PAX8, to 112-gene panels [95]. The small seven-gene panel includes the most common gene alterations in thyroid nodules and has a high specificity. Therefore, it may be useful as a rule-in test. According to a recent prospective study, this test can significantly increase the probability of cancer in mutated undetermined thyroid nodules, with a specificity of 95% and a PPV of 67% [98]. Commercially available panels for molecular testing of indeterminate thyroid nodules include Afirma GSC, Thyroseq v3, and ThyGeNEXT/ThyraMIR. Afirma GSC combines next-generation RNA sequencing and small genomic alterations that are not identifiable with standard methods. It is used mainly as a rule-out test with a high NPV of 96%, while the sensitivity, specificity, and PPV are 68, 91, and 47%, respectively [99]. Thyroseq v3 is a panel of 112 genes with a high NPV of 97%, and the sensitivity, specificity, and PPV are 82, 94, and 66%, respectively, and is also applicable as a rule-out test [99]. ThyGeNEXT is a next-generation sequencing panel with the purpose of sequencing gene mutations (BRAF, NRAS, HRAS, KRAS, and PIK3CA) and mRNA fusions (RET/PTC1, RET/PTC3, and PPARG/PAX8) [95]. It is combined with a miRNA risk classifier ThyraMIR, which determines the expression of growth-promoting miRNA (miR-31, -146, -222, -375, -551) and growth-suppressing miRNA (miR-29, -138, -139, -155, -204) and increases its accuracy, demonstrating sensitivity, specificity, NPV, and PPV of 90, 93, 95, and 74%, respectively [99].

In terms of prognosis, a recent study demonstrated that tumors with BRAF-like profiles positively correlate with larger tumors, higher initial tumor stage, and presence of lateral neck metastasis, compared with the RAS-like profile tumors and non-BRAF/non-RAS-like tumors which included PAX8::PPARG fusion and DICER1, EIF11AX, PTEN, and IDH1 mutations [100].

In malignant thyroid nodules (Bethesda VI), BRAF^V600E^ mutation is detected in 64–76% of tumors, while TERT promoter mutations are detected in 11% of cases [101]. The presence of both mutations positively correlates with extrathyroidal extension, local and distant metastasis, tumor recurrence, and mortality [99], and predicts the development of radioiodine-refractory DTC in PTC patients [102].

## 5. Integrated Diagnostics of Thyroid Nodules

Even if thyroid nodules are very common, randomized clinical trials are scarce, likely due, almost partially, to the good prognosis of thyroid cancer. Instead, recommendations in clinical guidelines are largely based on observational studies and experts’ opinions. Thyroid nodules mostly derive from follicular thyroid cells, and benign nodules (unifocal or multifocal) are the most common thyroid lesions. Additionally, thyroid nodules may also occur and coexist in conditions such as subacute thyroiditis, autoimmune thyroiditis, and Graves’ disease, respectively. Thyroid cancer, however, occurs in 2–5% of thyroid nodules. Finally, infiltrative disorders, lymphoma, metastases from non-thyroid cancers, and paraganglioma can rarely result in a thyroid nodule. Factors related to an increased risk of cancer are summarized in Table 8.

As thyroid nodules are very frequent while thyroid cancers are rare and relatively indolent in most cases, the main challenge in approaching patients with thyroid nodules is to identify malignant lesions while avoiding inappropriate excess use of thyroid US, scintigraphy, FNAC, and surgery. Unfortunately, a significant lack of standardization in the preoperative characterization of thyroid nodules is reported in the literature and in guidelines, and, in turn, is commonly observed in clinical practice [103].

To further complicate the problem, a major proportion of nodules are currently detected during imaging examinations for non-thyroid issues or during so-called thyroid nodule screening. Notably, thyroid US examination is discouraged in patients without clinical evidence of thyroid enlargement or nodules, and thyroid incidentaloma, especially when <10 mm, should not be referred for additional examinations [104]. Unfortunately, such concepts are rarely incorporated into clinical practice and a plethora of non-significant nodules are detected, inducing fear and anxiety in our patients and a lot of inappropriate additional examinations [105]. Interestingly, Asian thyroid nodule practice has a more conservative approach in general, not only for indeterminate thyroid nodules [106] but also for papillary microcarcinoma [107,108].

Usually, patients experience cancer fears and anxiety during the evaluation of thyroid nodules. Accordingly, a “one-stop-shop” multidisciplinary approach represents an ideal solution. The objectives of a one-stop-shop diagnostic thyroid unit are to concentrate in one place and time all specialists required to provide a correct diagnosis and reach a clinical decision as soon as possible (ideally within 1 day). In any case, patients should be first examined by an experienced clinician (i.e., endocrinologist, nuclear medicine physician, endocrine surgeon) and a blood sample should be obtained for TSH measurement. Based on clinical examination and TSH results, most patients can be properly informed and reassured, and avoid further examinations.

Among patients with clinically relevant nodules and low-to-suppressed TSH levels, a thyroid scintigraphy with either Na[^99m^Tc]TcO_4_ or Na[^123^I]I should be ordered to detect autonomously functioning nodules and exclude them from FNAC, as malignancies are exceedingly rare in such cases. In other cases, US examination should be performed and interpreted/reported according to one of the available TI-RADS systems in order to standardize the selection of nodules that need further investigation with FNAC. All in all, FNAC will be necessary for the minority of patients with non-autonomous thyroid nodules and a high-risk TIRADS score. Cytopathology examinations should be reported according to the Bethesda System for Reporting Thyroid Cytopathology and molecular imaging should be considered in selected patients with indeterminate non-Hurtle cell cytology and no additional factors in favor of surgery (i.e., multinodular goiter). Proper patient–physician communication is pivotal before any step in order to clearly explain the pros and cons of a data procedure and its impact on clinical decisions.

**Figure 7:**
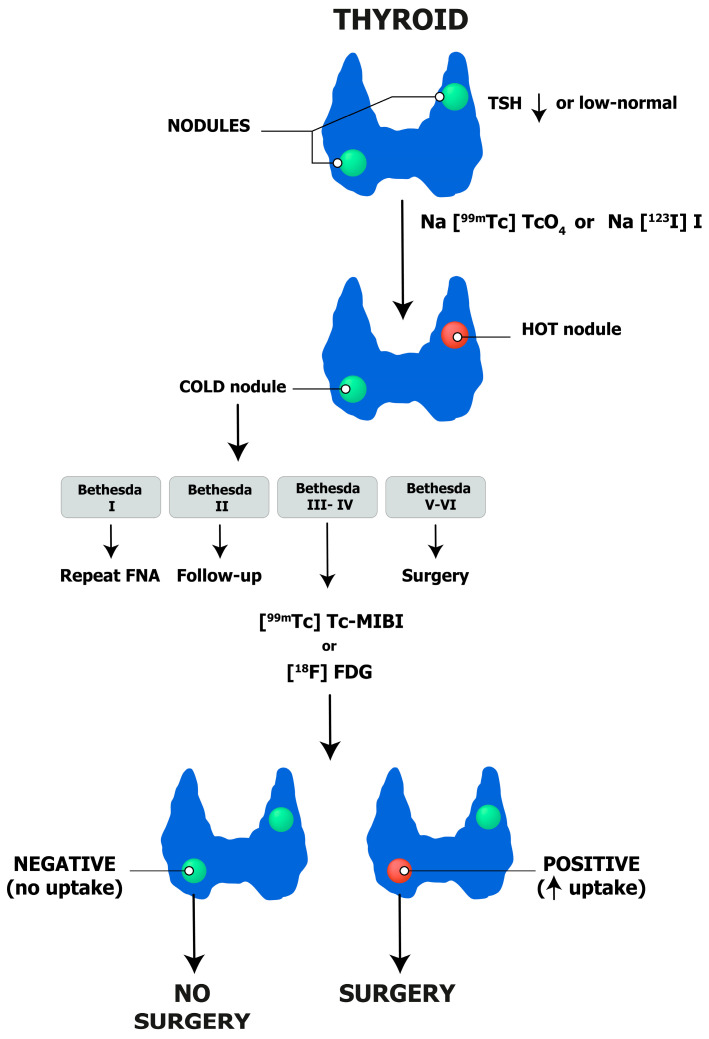
Thyroid nodules: integrated diagnostic flow-chart.

Finally, integrating different reports allows the coordinator physician to inform the patient about the diagnosis and formulate a tailored management plan (i.e., surgery, wait and see, thermal ablation). A diagnostic algorithm is proposed in Figure 7.

The implementation of this algorithm into clinical practice is mandatory to reassure patients regarding unnecessary therapeutic procedures and to identify those who need specific therapies. The value of thyroid US and its scoring systems, scintigraphy, FNAC, and molecular imaging is proven in many previously mentioned studies and requires further, more extensive, and wider integration into the national guidelines of each country to ensure widespread adoption and accessibility of integrated diagnostics of thyroid nodules.

## 6. Perspectives

The process presented above (i.e., clinical integration) is based on high-standing diagnostic methods and physicians’ experience in interpreting and integrating different data. This is a long process requiring time and resource investment and continuous education. Artificial intelligence (AI) and machine learning (ML) can support clinicians by providing them with additional insights not routinely available in clinical practice. The high potential of AI and ML can be exploited in “integrated diagnostics”, defined as the “convergence of imaging, pathology, and laboratory tests with advanced information technology” [109].

AI algorithms can be trained to analyze images from multiple modalities and detect phenotyping information that may not be obvious to the human eye. Lai and colleagues compared the diagnostic performance of artificial intelligence algorithms and radiologists with different experience levels in distinguishing benign and malignant TI-RADS 4 nodules where heterogeneity is observed in the malignancy rates. They enrolled 1117 pathologically defined TI-RADS 4 nodules and incorporated an independent external dataset of 125 TIRADS-4 nodules for testing purposes. Traditional feature-based machine learning models, deep convolutional neural network (DCNN) models, and a fusion model that integrated all prediction outcomes were used to score benign and malignant TI-RADS 4 nodules. A fivefold cross-validation approach was employed, and the diagnostic performance of each model and radiologist was compared. Briefly, in the external test, the area under the receiver operating characteristic curve (AUROC) of the three tested DCNN models ranged from 0.852 to 0.856, respectively. These values were higher than those of the three traditional ML models (0.767–0.709), respectively, and higher than that of an experienced radiologist (0.815). The fusion diagnostic model developed by the authors, with an AUROC of 0.880, outperformed the experienced radiologist in diagnosing TI-RADS 4 nodules [110]. These results demonstrate that automated clinical decision support significantly improves diagnostic accuracy, i.e., sensitivity, specificity, and AUC, and minimizes interpretation times and inter-reader variability. AI is also used in the analysis of genomic data [111].

AI algorithms analyze large amounts of data and identify genetic variations that may be associated with cancers or other common non-communicable diseases [112] or assist in the interpretation of laboratory test results, such as pathology reports, and other diagnostic data. As an example, longitudinal data analysis centered on collecting and analyzing longitudinal data, specifically cluster analysis, already showed clinical usefulness in patients with chronic renal failure for determining the required dialysis frequency [113].

**Figure 8:**
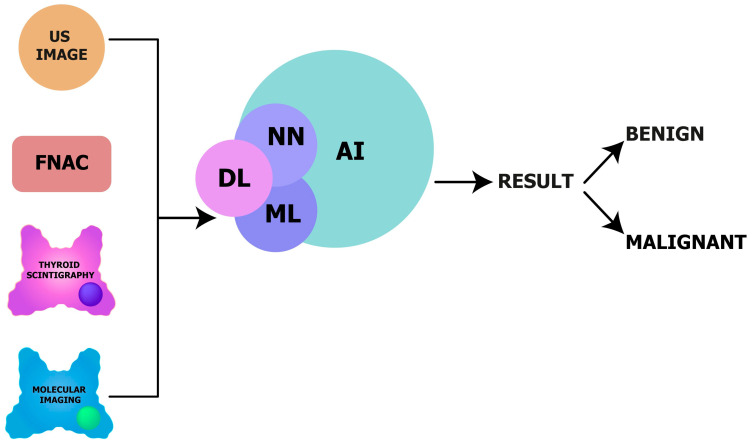
Potential role of deep learning, neural networks, machine learning, and artificial intelligence in thyroid nodule management. Legend: US image, ultrasonography image; FNAC, fine-needle aspiration cytology; DL, deep learning; NN, neural networks; ML, machine learning; AI, artificial intelligence.

Digital image analysis in pathology can identify and quantify specific cell types for quickly and accurately evaluating histological features, morphological patterns, and biologically relevant regions of interest [114]. Overall, AI is playing a growing role in the diagnosis and management of diseases through integrated diagnostics, and appears promising even in endocrine and thyroid diseases [115] (Figure 8).

However, some limitations should be considered, including the need for large amounts of data to train AI algorithms and the need for more research to validate the use of AI. Moreover, the broad implementation of AI and integrated diagnostics requires central organizations (i.e., national or international level) to ensure common structures and methodologies, standards, and data safety.

An extensive discussion of such issues is out of the scope of our present paper; our readers are directed to a recent review by Ali MA and Mohammed MA where the inherent limitations of AI in analyzing omics data (i.e., preprocessing, datasets, validation of models, testbed applications) are illustrated while potential solutions are proposed [116].

Future research integrating deep learning, ML, and AI on US images, FNAC, thyroid scintigraphy, and specific molecular imaging is required to determine their value in discriminating benign from malignant thyroid nodules, especially in cases of indeterminate thyroid nodules where current molecular imaging is more powerful as a rule-out than rule-in test.

## 7. Limitations

The majority of the reported data came from retrospective studies, with inherent methodological limitations. However, the roles of US, FNAC, and thyroid scintigraphy are consolidated in clinical practice and defined in clinical guidelines. Moreover, prospective, and even randomized, studies are available for more recent molecular imaging methods as well as cost-effectiveness analysis. Accordingly, the proposed modalities and their use seem to be adequately supported by the current literature.

## 8. Conclusions

The current state of diagnostic medicine is well presented by the “silo metaphor”, where radiology, laboratory medicine, and pathology are conceptually separate diagnostic approaches. On the other hand, progress in understanding biochemical–biological–structural interplays in human diseases, compounded with technological advances, is generating relevant multidisciplinary convergences, leading the way for a new frontier called integrated diagnostics. Thyroid nodules are commonly encountered in clinical practice and their diagnosis and therapy typically involve different specialists using different tools. Accordingly, thyroid nodules represent an ideal condition to develop and test integrated diagnostics: an appropriate integration of laboratory, imaging, and pathology data is essential to refine our diagnosis, reassure most patients with non-dangerous nodules, and quickly identify those requiring specific therapies. Furthermore, bioinformatics and computer sciences will change our current management of clinical data, allowing more personalized and tailored diagnostic approaches.

The main advantages of our proposed diagnostic flow-chart are *i.* the reduction in the number of nodules requiring any imaging procedure through use of a careful clinical examination; *ii.* the reduction in FNAC procedures by using TIRADS criteria and (selectively) functional thyroid scintigraphy; and *iii.* the reduction in inappropriate diagnostic surgeries in patients carrying cytologically indeterminate nodules through selective use of new molecular imaging procedures.

Looking forward, we believe studies concerning the management of thyroid incidentalomas are urgently needed to inform radiological reports and, hopefully, spare most patients from any further investigation. In addition, the proper use of thyroid US (i.e., avoiding “screening”) should be more effectively supported by clinical societies. In such context, the selective use of more advanced methods in patients with clinically relevant, well-characterized nodules will be useful in avoiding unjustified surgeries and will be cost-effective as well.

## Figures and Tables

**Table 1:** Thyroid ultrasound: clinical indications.

Indications
■ To evaluate thyroid nodules and differentiate between benign and malignant ones
■ To evaluate diffuse changes in the thyroid
■ To differentiate thyroid nodules from cervical cysts, thyroglossal duct, and cervical masses
■ To monitor patients with thyroid malignancies and detect recurrent/metastatic disease
■ To guide interventional procedures (FNAC, PEI, TA)

Legend: FNAC, fine-needle aspiration cytology; PEI, percutaneous ethanol injection; TA, thermal ablation.

**Table 2:** EU-TIRADS categories, risks of malignancy, and recommendations [46].

Category	US Features	Malignancy Risk, %	Recommendations
EU-TIRADS 1: Normal	No nodules	None	None
EU-TIRADS 2: benign	Pure cyst, Entirely spongiform	0	No FNA required (unless for therapeutic purposes/to relieve compression)
EU-TIRADS 3: low risk	Ovoid, smooth isoechoic/hyperechoic No features of high suspicion	2–4	>20 mm FNA
EU-TIRADS 4: intermediate risk	Ovoid, smooth, mildly hypoechoic No features of high suspicion	6–17	>15 mm FNA
EU-TIRADS 5: high risk	At least 1 of the following features of high suspicion: – Irregular shape – Irregular margins – Microcalcifications – Marked hypoechogenicity (and solid)	26–87	>10 mm FNA, <10 mm: consider FNA or active surveillance

Legend: EU-TIRADS, European Thyroid Imaging Reporting and Data System; US, ultrasound.

**Table 3:** ATA sonographic patterns, estimated risk of malignancy, and fine-needle aspiration guidance for thyroid nodules [18].

Sonographic Pattern	US Features	Estimated Risk of Malignancy, %	FNA Size Cutoff (Largest Dimension)
High suspicion	Solid hypoechoic nodule or solid hypoechoic component of a partially cystic nodule *with* one or more of the following features: irregular margins (infiltrative, micro-lobulated), microcalcifications, taller-than-wide shape, rim calcifications with small extrusive soft tissue component, evidence of extrathyroidal extension	>70–90	Recommend FNA at ≥1 cm
Intermediate suspicion	Hypoechoic solid nodule with smooth margins *without* microcalcifications, ETE, or taller-than-wide shape	10–20	Recommend FNA at ≥1 cm
Low suspicion	Isoechoic or hyperechoic solid nodule, or partially cystic nodule with eccentric solid areas, *without* microcalcification, irregular margin or ETE, or taller-than-wide shape	5–10	Recommend FNA at ≥1.5 cm
Very low suspicion	Spongiform or partially cystic nodules *without* any of the sonographic features described in low-, intermediate-, or high-suspicion patterns	<3	Consider FNA at ≥2 cm Observation without FNA is also a reasonable option
Benign	Purely cystic nodules (no solid component)	<1	No biopsy

Legend: US, ultrasound; FNA, fine-needle aspiration; ETE, extrathyroidal extension.

**Table 4:** Ultrasound-based recommendations for fine-needle aspiration cytology: (a) Biopsy recommendations of EU TI-RADS [46]. (b) Biopsy recommendations of ACR TI-RADS guidelines [45]. (c) Biopsy recommendations of ATA guidelines [18].

(**a**)			
**Category**		**Malignancy Risk, %**	**Recommendations**
EU-TIRADS 1: Normal		None	None
EU-TIRADS 2		benign, malignancy risk 0%;	No FNA required (unless for therapeutic purposes/to relieve compression)
EU-TIRADS 3		low risk, malignancy risk 2–4%	>20 mm FNA
EU-TIRADS 4		intermediate risk, malignancy risk 6–17%	>15 mm FNA
EU-TIRADS 5		high risk, malignancy risk 26–87%	>10 mm FNA, <10 mm: consider FNA or active surveillance
(**b**)			
**Category**	** Points **	**Malignancy Risk, %**	**Recommendations**
TR1:	0 points	benign (aggregate risk level 0.3%);	No FNA required
TR2:	2 points	not suspicious (aggregate risk level 1.5%);	No FNA required
TR3:	3 points	mildly suspicious (aggregate risk level 4.8%);	≥25 mm FNA
TR4:	4–6 points	moderately suspicious (aggregate risk level 5.9–12.8%);	≥15 mm FNA
TR5:	7 points or more	highly suspicious (aggregate risk level 20.8–68.4% for 10 points).	≥10 mm FNA,
(**c**)			
**Category**		**Malignancy Risk, %**	**Recommendations**
Benign		Risk level < 1%	No FNA required
Very low suspicion		Risk level < 3%	Consider FNA at ≥2 cm Observation without FNA is also a reasonable option
Low suspicion		Risk level 5–10%	Recommend FNA at ≥1.5 cm
Intermediate suspicion		Risk level 10–20%	Recommend FNA at ≥1 cm
High suspicion		Risk level > 70–90%	Recommend FNA at ≥1 cm

**Table 5:** The 2017 Bethesda System for Reporting Thyroid Cytopathology: implied risk of malignancy and recommended clinical management [93].

Diagnostic Category	Risk of Malignancy if NIFTP ≠ CA (%)	Risk of Malignancy if NIFTP = CA (%)	Usual Management
I. Non-diagnostic or unsatisfactory	5–10	5–10	Repeat FNA with ultrasound guidance
II. Benign	0–3	0–3	Clinical and sonographic follow-up
III. Atypia of undetermined significance or follicular lesion of undetermined significance	6–18	∼10–30	Repeat FNA, molecular testing, or lobectomy
IV. Follicular neoplasm or suspicious for a follicular neoplasm	10–40	25–40	Molecular testing, lobectomy
V. Suspicious for malignancy	45–60	50–75	Near-total thyroidectomy or lobectomy
VI. Malignant	94–96	97–99	Near-total thyroidectomy or lobectomy

**Table 6:** Comparison between 2014 Italian SIAPEC-AIT classification, 2017 Bethesda, and 2016 UK RCPath reporting system for thyroid cytology.

RCPath	Bethesda	Italian SIAPEC-AIT
Thy1 Non-diagnostic for cytological diagnosis Thy1c Non-diagnostic for cytological diagnosis—cystic lesion	I. Non-diagnostic or unsatisfactory II. Virtually acellular specimen III. Other (obscuring blood, clotting artifact, etc.) IV. Cyst fluid only	TIR1 Non-diagnostic TIR1c Non-diagnostic cystic
Thy2 Non-neoplastic Thy2c Non-neoplastic, cystic lesion	Consistent with a benign follicular nodule (includes adenomatoid nodule, colloid nodule, etc.). Consistent with lymphocytic (Hashimoto) thyroiditis in the proper clinical context Consistent with granulomatous (subacute) thyroiditis	TIR2 Non-malignant
Thy3a Neoplasm possible—atypia/non-diagnostic	III. Atypia of undetermined significance or follicular lesion of undetermined significance	TIR3A Low-risk indeterminate lesion (LRIL)
Thy3f Neoplasm possible, suggesting follicular neoplasm	IV. Follicular neoplasm or suspicious for a follicular neoplasm Specify if Hürthle cell (oncocytic) type	TIR3B High-risk indeterminate lesion (HRIL)
Thy4 Suspicious of malignancy	V. Suspicious for malignancy	TIR4 Suspicious of malignancy
Thy5 Malignant	VI. Malignant	TIR5 Malignant

**Table 7:** “Indeterminate” diagnostic categories: comparison of risk of malignancy (ROM) and clinical management between Italian, Bethesda, and British classifications.

2014 Italian SIAPEC-AIT		2017 Bethesda		2016 RCPath Classification	
Diagnostic category (ROM %)	Management	Diagnostic category (ROM %)	Management	Diagnostic category (ROM %)	Management
TIR 3A Low-risk indeterminate lesion (12–22%)	Clinical follow up/Repeat FNA	III. AUS/FLUS (10–30%)	Repeat FNA/Molecular testing or lobectomy	Thy 3a Neoplasm possible – atypia/non-diagnostic (25%)	Multidisciplinary assessment
TIR 3B High-risk indeterminate lesion (30–55%)	Surgery	IV. Follicular neoplasm/suspicious follicular neoplasm (25–40%)	Molecular testing, lobectomy	Thy 3f Neoplasm possible, suggesting follicular neoplasm (31%)	Multidisciplinary assessment

Legend: ROM: risk of malignancy; AUS: atypia of undetermined significance; FLUS: follicular lesion of undetermined significance.

**Table 8:** Thyroid nodules: factors increasing the risk of cancer.

History	■ Childhood irradiation (head and neck) ■ Fallout from ionizing radiation ■ Radiation exposure ■ Family history of thyroid cancer ■ Enlarging nodule or rapid nodule growth ■ Male sex ■ Age less than 20 or more than 70 years
Clinical examination	■ Neck lymphadenopathy ■ Hard nodule ■ Nodule fixed to surrounding tissues ■ Hoarseness
Clinical investigations	■ Suspicious ultrasound features ■ High-risk TI-RADS classes ■ Largest diameter > 40 mm ■ Thyroid scintigraphy (Na[^99m^Tc]TcO_4_ or Na[^123^I]I): not hot ■ Thyroid scintigraphy ([^99m^Tc]Tc-MIBI): high uptake, slow washout ■ [^18^F]FDG -avid (PET/CT) ■ Serum calcitonin > 100 pg/mL (MTC)

Legend: TI-RADS, Thyroid Imaging-Reporting and Data System; Na[^99m^Tc]TcO_4_, ^99m^Tc-pertechnetate; Na[^123^I]I, iodine-123; PET/CT, positron emission tomography/computed tomography; MTC, medullary thyroid cancer.

## Data Availability

Data sharing not applicable.
